# Hearing loss, hearing aid use, and subjective memory complaints: Results of the HUNT study in Norway

**DOI:** 10.3389/fneur.2022.1094270

**Published:** 2023-01-11

**Authors:** Shahram Moradi, Bo Engdahl, Aud Johannessen, Geir Selbæk, Lisa Aarhus, Gro Gade Haanes

**Affiliations:** ^1^Department of Health, Social and Welfare Studies, Faculty of Health and Social Sciences, University of South-Eastern Norway, Porsgrunn, Norway; ^2^Department of Physical Health and Ageing, Norwegian Institute of Public Health, Oslo, Norway; ^3^Department of Health, Social and Welfare Studies, Faculty of Health and Social Sciences, University of South-Eastern Norway, Horten, Norway; ^4^Norwegian National Centre for Ageing and Health, Vestfold Hospital Trust, Tønsberg, Norway; ^5^Faculty of Medicine, Institute of Clinical Medicine, University of Oslo, Oslo, Norway; ^6^Geriatric Department, Oslo University Hospital, Oslo, Norway; ^7^Department of Occupational Medicine and Epidemiology, National Institute of Occupational Health, Oslo, Norway; ^8^Medical Department, Diakonhjemmet Hospital, Oslo, Norway; ^9^Department of Nursing and Health Sciences, Faculty of Health and Social Sciences, University of South-Eastern Norway, Horten, Norway

**Keywords:** hearing loss, subjective memory complaints, hearing aid use, short-term (working) memory, long-term memory

## Abstract

**Objective:**

This study aimed to explore the association between hearing loss severity, hearing aid use, and subjective memory complaints in a large cross-sectional study in Norway.

**Methods:**

Data were drawn from the fourth wave of the Trøndelag Health Study (HUNT4 Hearing, 2017–2019). The hearing threshold was defined as the pure-tone average of 0.5, 1, 2, and 4 kHz in the better ear. The participants were divided into five groups: normal hearing or slight/mild/moderate/severe hearing loss. Subjective self-reported short-term and long-term memory complaints were measured by the nine-item Meta-Memory Questionnaire (MMQ). The sample included 20,092 individuals (11,675 women, mean age 58.3 years) who completed both hearing and MMQ tasks. A multivariate analysis of variance (adjusted for covariates of age, sex, education, and health cofounders) was used to evaluate the association between hearing status and hearing aid use (in the hearing-impaired groups) and long-term and short-term subjective memory complaints.

**Results:**

A multivariate analysis of variance, followed by univariate ANOVA and pairwise comparisons, showed that hearing loss was associated only with more long-term subjective memory complaints and not with short-term subjective memory complaints. In the hearing-impaired groups, the univariate main effect of hearing aid use was only observed for subjective long-term memory complaints and not for subjective short-term memory complaints. Similarly, the univariate interaction of hearing aid use and hearing status was significant for subjective long-term memory complaints and not for subjective short-term memory complaints. Pairwise comparisons, however, revealed no significant differences between hearing loss groups with respect to subjective long-term complaints.

**Conclusion:**

This cross-sectional study indicates an association between hearing loss and subjective long-term memory complaints but not with subjective short-term memory complaints. In addition, an interaction between hearing status and hearing aid use for subjective long-term memory complaints was observed in hearing-impaired groups, which calls for future research to examine the effects of hearing aid use on different memory systems.

## 1. Introduction

Hearing loss is one of the most common sensory disabilities in older adults. It adversely affects language understanding. Hearing loss is also a risk factor for cognitive decline [e.g., (1)] and dementia (2, 3). Furthermore, hearing loss impacts neural systems involved in the processing of speech signals [e.g., (4)], which leads to structural and functional changes in the brain (5, 6).

The extent to which hearing loss affects the human memory system has been less extensively investigated. The human memory system consists of multiple theoretical systems, including short-term memory (the capacity for retention of a small amount of information for a short period of time) and long-term memory. Long-term memory is typically categorized into declarative memory (conscious, explicit recollection or recognition of events and facts) and non-declarative memory (unconscious, implicit knowledge of habits, skills, routines, and procedures) (7, 8).

There are objective and subjective methods to measure memory in humans. Objective memory refers to the recall of items, personal experiences, and general knowledge. On the other hand, subjective memory complaints refer to people's self-evaluation of memory dysfunction and individuals' awareness of memory failure in the absence of objective memory impairment (9). One advantage of using subjective self-reported memory scales is their independence from participants' sensory functioning which is likely to affect performance in objective memory tasks. It should be noted that objective tests of memory function can be independent of sensory function when the method of testing is not dependent on the sensory function of interest. For example, the outcomes of visual tests of memory function are probably unaffected by hearing loss status when participants meet the criteria of normal or corrected to normal vision. With regard to objective memory assessment, prior research showed mixed results regarding the effects of hearing loss on objective memory tasks. For example, Rönnberg et al. (10) by using memory tests that were independent of the hearing functioning of participants found that hearing loss adversely affected semantic and episodic long-term memory but not short-term memory. Using data from the United Kingdom Biobank cohort study, Rönnberg et al. (11) reported that hearing loss negatively affected both long-term and short-term visuospatial memory tasks. Loughrey et al. (12), using data from the Irish Longitudinal Study of Aging, found no direct effect of subjective self-reported hearing difficulties on episodic long-term memory performance. Regarding subjective memory complaints, Curhan et al. (13, 14), using data from longitudinal cohort studies, investigated the extent to which self-reported hearing loss affected subjective memory concerns in men and women. The results showed that hearing loss was linked to increased complaints regarding subjective memory function in both men and women. In addition, more self-reported hearing loss was associated with greater subjective memory complaints. Jayakody et al. (15) studied the association between subjective memory complaints and peripheral hearing and central auditory processing in a sample of individuals aged 45–85 years old with average hearing thresholds (for the better hearing ear), at frequencies of 0.5, 1, 2, and 4 kHz (4PTA), of <40 dBHL. The results showed that, compared with people with no subjective memory complaints (*n* = 34), those with subjective memory complaints (*n* = 61) performed poorly only on a sentence-identification-in-noise test. No significant differences were observed between groups in terms of 4PTA and quick speech-in-noise test results. The association between speech-in-noise testing and memory complaints in Jayakody et al. (15) is not surprising as perceiving speech in noise demands a greater cognitive load. Individuals with memory complaints could have reduced cognitive capacities for processing speech signals in noisy conditions. In addition, the lack of association between subjective memory complaints and PTA4 is not surprising given the hearing was better than 40 dBHL (normal to mild hearing loss).

Currently, sound amplification with a hearing aid is the most common rehabilitative treatment to enhance speech perception ability in people with hearing loss. Modern digital hearing aids greatly enhance speech perception in people with hearing loss; however, hearing aid users still lag behind people with normal hearing when perceiving speech signals in the absence of a supportive semantic context [see (16, 17)]. Literature regarding hearing aids and cognitive function in people with hearing loss is not conclusive. Some studies have shown no difference between hearing-aid users and non-hearing-aid users in terms of cognitive function [e.g., (18–21)]. However, Maharani et al. (22) reported that hearing aid use slowed down episodic memory decline. In addition, Rönnberg et al. (11) reported that hearing aid use had a small positive effect on short-term visuospatial memory but not on long-term visuospatial memory. Karawani et al. (23) showed that using a hearing aid for longer than 6 months was associated with neuroplastic changes in the brain and increased working memory capacity. No effect of hearing aid use was observed on processing speed and attentional capacity.

Using a larger sample than prior studies, the present study aimed to investigate the association between hearing loss, hearing aid use, and subjective memory complaints, after controlling for confounders including age, sex, education, and health variables, using data from the HUNT4 Hearing in Norway. No study has yet examined the extent to which hearing aid use affects subjective memory complaints in people with hearing loss.

## 2. Materials and methods

### 2.1. Participants

The HUNT4 Hearing, which was a part of HUNT4, was conducted in Nord-Trøndelag County in Norway [see (24) for more information about HUNT4 Hearing]. All residents aged 20 years and over were invited to take part. The participation rate for HUNT4 Hearing was approximately 43%, and 28,302 completed the audiometric tasks. The baseline sample in this study consisted of 20,092 individuals (11,675 women, mean age 58.3 years) who completed the meta-memory questionnaire (MMQ). The participants signed an informed consent form for their participation in HUNT4 Hearing. The study was approved by the regional committee for medical and health research ethics and the Norwegian Data Protection Authority (23178 HUNT Hørsel).

### 2.2. Hearing status

Engdahl et al. (25) provided detailed information about the hearing screening of participants and measuring audiometric thresholds in the HUNT4 Hearing. In short, several teams were involved in collecting data for this project. Each team had a trained audiologist and two trained assistants. A questionnaire was used to evaluate subjective hearing loss, tinnitus, hearing aid use, and other risk factors for causing hearing loss. Then, the participants underwent otoscopy and pure-tone audiometry. The pure-tone audiometric thresholds were measured using Interacoustics audiometers (type AD629) in semiportable, dismountable sound booths (IAC Moduline System, 102 mm thick, 1,450 × 1,450 × 2,100 mm^3^).

We defined the hearing status of participants based on hearing thresholds for the pure-tone average of four frequencies (500, 1,000, 2,000, and 4,000 Hz, or 4PTA) in the better hearing ear: normal hearing (4PTA hearing threshold, ≤15 dB), slight hearing loss (4PTA, 16–25 dB), mild hearing loss (4PTA, 26–40 dB), moderate hearing loss (4PTA, 41–55 dB), and severe hearing loss (4PTA, ≥56 dB).

### 2.3. Meta-Memory Questionnaire

In the HUNT study, the nine-item MMQ was used to examine participants' subjective memory complaints. The MMQ was initially developed for a Nordic study on aging and health, to assess memory function in a single score (26). The MMQ comprises nine items about memory complaints. The first two items ask about memory function in general: “(1) Do you have problems with your memory?” and “(2) Has your memory changed since you were younger?” The response categories are “no,” “yes, sometimes,” and “yes, a lot.” The next seven items ask about specific memory functions, starting with the question “do you have problems remembering”: “(3) that happened few minutes ago,” “(4) names of other people,” “(5) dates,” “(6) to carry out planned activities,” “(7) that happened a few days ago,” “(8) that happened years ago,” and “(9) keeping track of a conversation.” The possible responses for these seven items are “never,” “sometimes,” and “often.”

Almkvist et al. (27), after conducting a principle component factor analysis on the MMQ in the third wave of the HUNT cohort study (2006–2008, HUNT3), revealed that items 1, 2, 4, 5, and 8 were related to declarative long-term memory complaints and items 3, 6, 7, and 9 were related to short-term memory complaints. The scoring for questions 1 and 2 was as follows: 0 = no, 1 = yes, sometimes, and 2 = yes, a lot. For questions 3–9, the scoring was as follows: 1 = never, 2 = sometimes, and 3 = often. The total score was calculated by summing the scores for each subjective memory component (the range was from 3 to 13 for long-term subjective memory complaints and 4–12 for short-term memory subjective memory complaints). We conducted a preliminary factor analysis using data from HUNT4. The results corroborated the findings by Almkvist et al. (27), as factor analysis revealed two main factors: short-term memory complaints (items 3, 6, 7, and 9) and long-term memory complaints (items 1, 2, 4, 5, and 8).

### 2.4. Statistical analysis

SPSS version 28 statistical software was used to analyze the data. A multivariate analysis of variance (MANOVA) with follow-up univariate analyses was performed to assess the effect of hearing status (normal hearing and various types of hearing loss) on long-term memory and short-term memory complaints. Pairwise comparisons were performed using the Bonferroni method. Wilks' lambda statistic was used to assess multivariate significance. The same MANOVA was also used to determine the effects of hearing loss severity and hearing aid use on subjective long-term memory and short-term memory complaints. Analyses were adjusted for the covariates of sex, age, education, stroke, diabetes, smoking, and hospital admission for a head injury. As the relationships between age and hearing and age and cognition are nonlinear, we categorized participants' age into different groups with 10-year intervals and treated age as a fixed factor in the analysis. All the other covariates were also categorical but treated as linear in the analyses. Missing data were listwise deleted in this study. The number of participants with missing data for each covariate was as follows: sex: *n* = 0; age: *n* = 13; education: *n* = 151; stroke: *n* = 1,312; diabetes: *n* = 463; smoking: *n* = 146; and hospital admission for a head injury: *n* = 2,670.

## 3. Results

Table 1 shows baseline sample characteristics stratified by hearing status. Because of missing data, the final sample was reduced to 16,141 participants (mean age at entry 57.7 years, 9,175 women). As Table 1 shows, individuals with moderate/severe degrees of hearing loss use hearing aids more than individuals with slight/mild degrees of hearing loss. In addition, participants with moderate/severe degrees of hearing loss were slightly older than participants with moderate/mild degrees of hearing loss and participants with normal hearing.

**Table 1:** Sample characteristics stratified by the hearing status of participants 463 (*n* = 16,141).

**Hearing status**	**Average Age (standard deviation)**	**Sex (%)**	**Average PTA4 (standard deviation)**	**Average subjective long-term memory complaints (standard deviation)**	**Average subjective short-term memory complaints (standard deviation)**	**Using hearing aid? (%)**	**Stroke? (%)**	**Hospital admission for head injury (%)**	**Diabetes? (%)**	**Smoking habits (%)**	**Educational level (%)**
Normal hearing	47.20 (14.99)	Females: 62 Males: 38	7.06 (6.18)	6.92 (2.04)	5.52 (1.78)		Yes (2) No (98)	Yes (7) No (92) I do not know (1)	Yes (4) No (96)	Never smoked (44) Former occasional smoker (10) Former daily smoker (36) Smoking occasionally (1) Daily smoker (8)	Primary (19) Secondary (31) Tertiary (49)
Slight hearing loss	65.15 (10.53)	Females: 51 Males: 49	23.06 (4.68)	7.29 (1.92)	5.67 (1.94)	Yes (8) No (92)	Yes (5) No (95)	Yes (7) No (92) I do not know (1)	Yes (10) No (90)	Never smoked (35) Former occasional smoker (6) Former daily smoker (48) Smoking occasionally (0.5) Daily smoker (10)	Primary (35) Secondary (31) Tertiary (35)
Mild hearing loss	71.13 (9.96)	Females: 48 Males: 52	35.12 (5.76)	7.45 (1.99)	5.75 (1.95)	Yes (32) No (68)	Yes (7) No (93)	Yes (6) No (93) I do not know (1)	Yes (11) No (89)	Never smoked (34) Former occasional smoker (5) Former daily smoker (53) Smoking occasionally (0.4) Daily smoker (8)	Primary (43) Secondary (26) Tertiary (31)
Moderate hearing loss	76.58 (9.21)	Females: 60 Males: 40	49.68 (5.94)	7.70 (2.00)	6.13 (2.06)	Yes (73) No (27)	Yes (9) No (91)	Yes (8) No (91) I do not know (1)	Yes (12) No (88)	Never smoked (35) Former occasional smoker (3) Former daily smoker (56) Smoking occasionally (0.3) Daily smoker (6)	Primary (47) Secondary (28) Tertiary (25)
Severe hearing loss	77.07 (12.75)	Females: 40 Males: 60	69.05 (11.09)	7.61 (2.22)	5.98 (2.16)	Yes (92) No (8)	Yes (10) No (90)	Yes (8) No (90) I do not know (2)	Yes (18) No (82)	Never smoked (39) Former occasional smoker (3) Former daily smoker (47) Smoking occasionally (0.6) Daily smoker (11)	Primary (44) Secondary (27) Tertiary (29)

**Figure 1:**
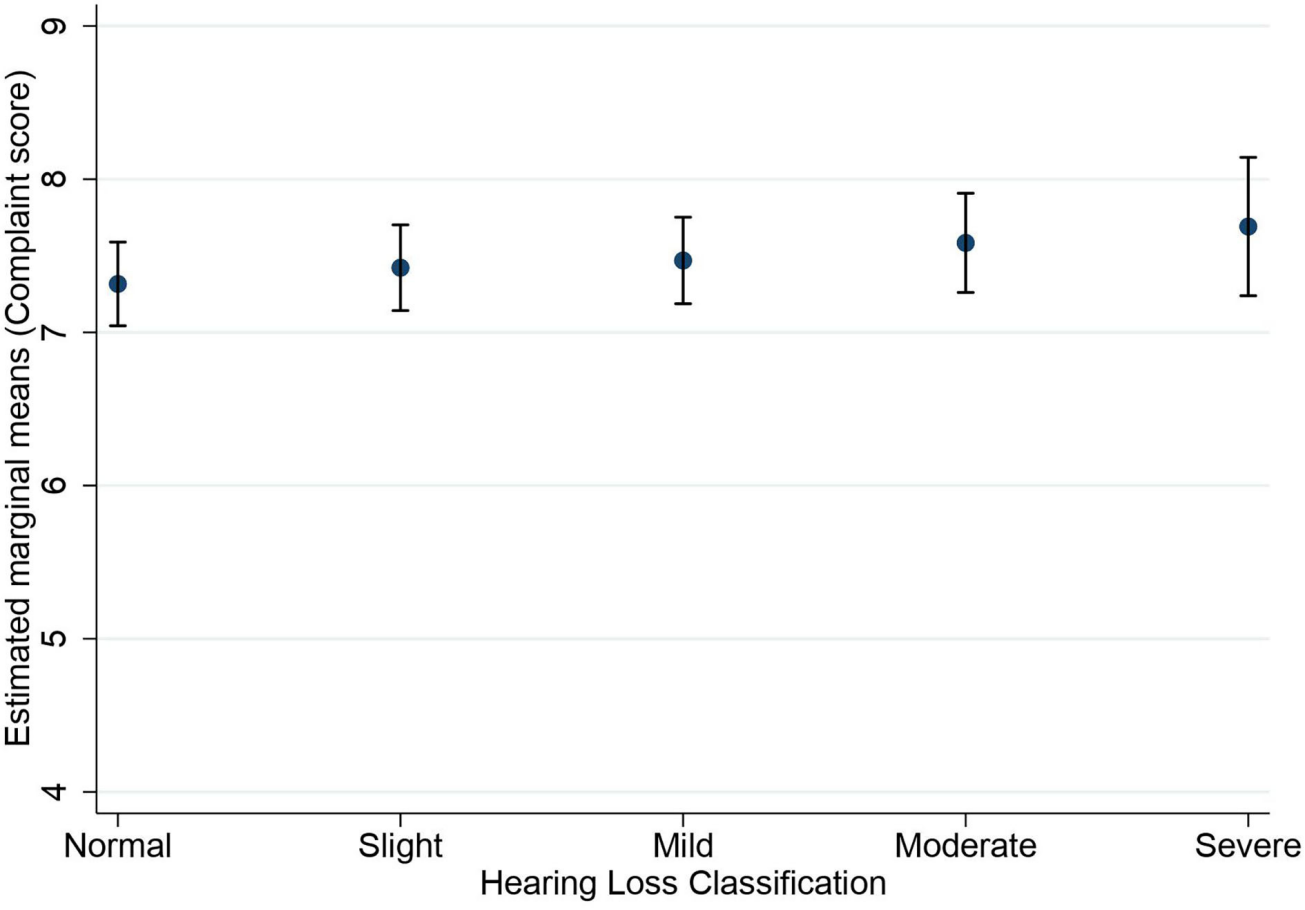
Estimated marginal means of subjective long-term memory complaints as a function of hearing status. Estimates are averaged over the levels of age at the means of the covariates sex, education, stroke, diabetes, smoking, and hospital admission for a head injury. The error bars indicate 95% confidence intervals.

**Figure 2:**
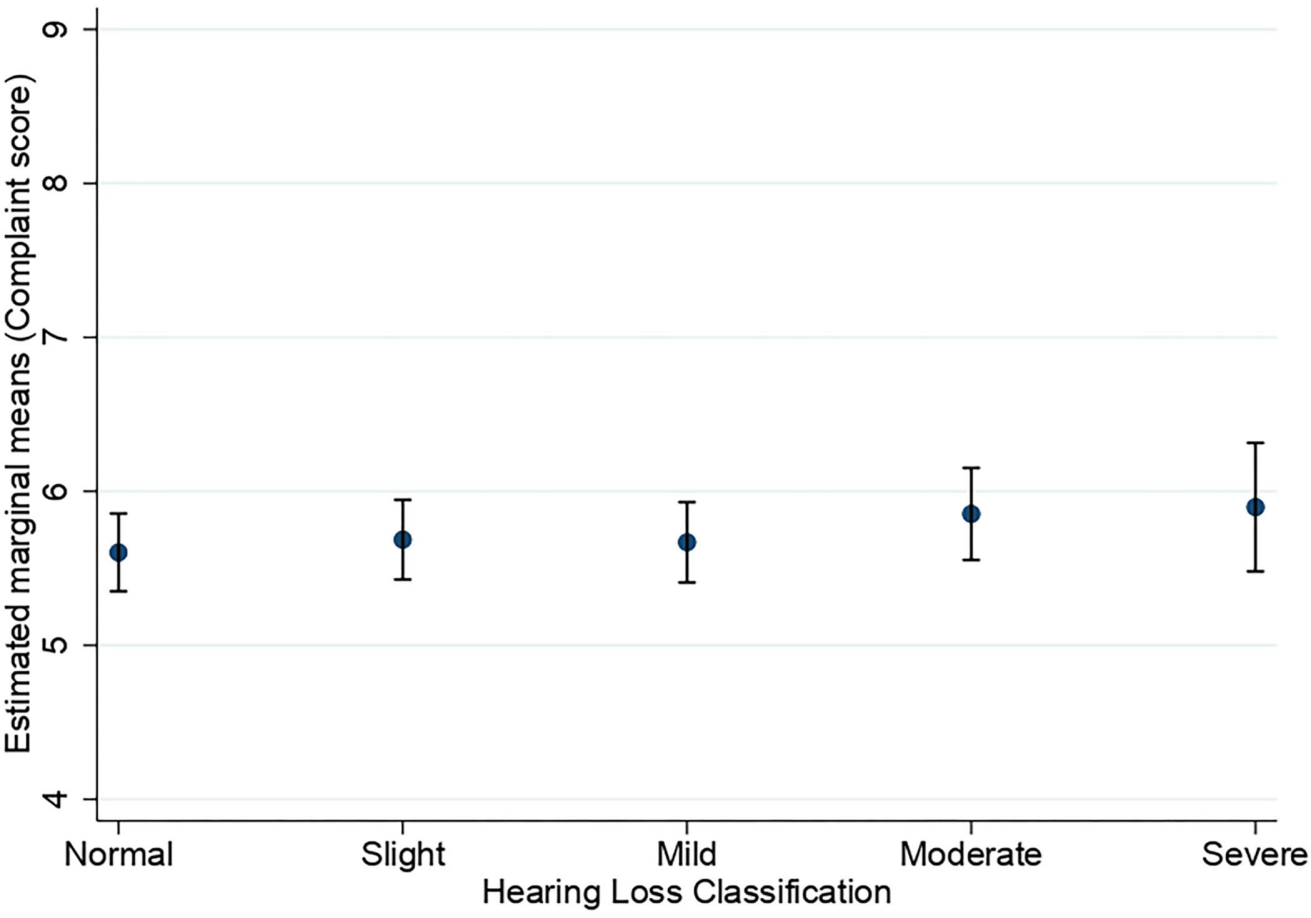
Estimated marginal means of subjective short-term memory complaints as a function of hearing status. Estimates are averaged over the levels of age at the means of the covariates sex, education, stroke, diabetes, smoking, and hospital admission for a head injury. The error bars indicate 95% confidence intervals.

Figures 1, 2 show the estimated marginal means for subjective long-term and short-term memory complaints as a function of hearing status. A two-way MANOVA analysis showed the multivariate main effect for hearing status was marginally insignificant (Wilk's λ = 0.999, *F*_(8,32,214)_ = 1.88, *p* = 0.058]. However, subsequent univariate ANOVAs revealed a main effect of hearing status for the subjective long-term memory complaints [*F*_(4,16,108)_ = 2.93, *p* = 0.020, η^2^ = 0.001] and not for subjective short-term memory complaints [*F*_(4,16,108)_ = 1.97, *p* = 0.10]. Bonferroni-adjusted pairwise comparisons in subjective long-term memory showed that the normal hearing group (*M* = 6.96) reported significantly fewer complaints than the slight hearing loss group only (*M* = 7.24, *p* = 0.011).

**Figure 3:**
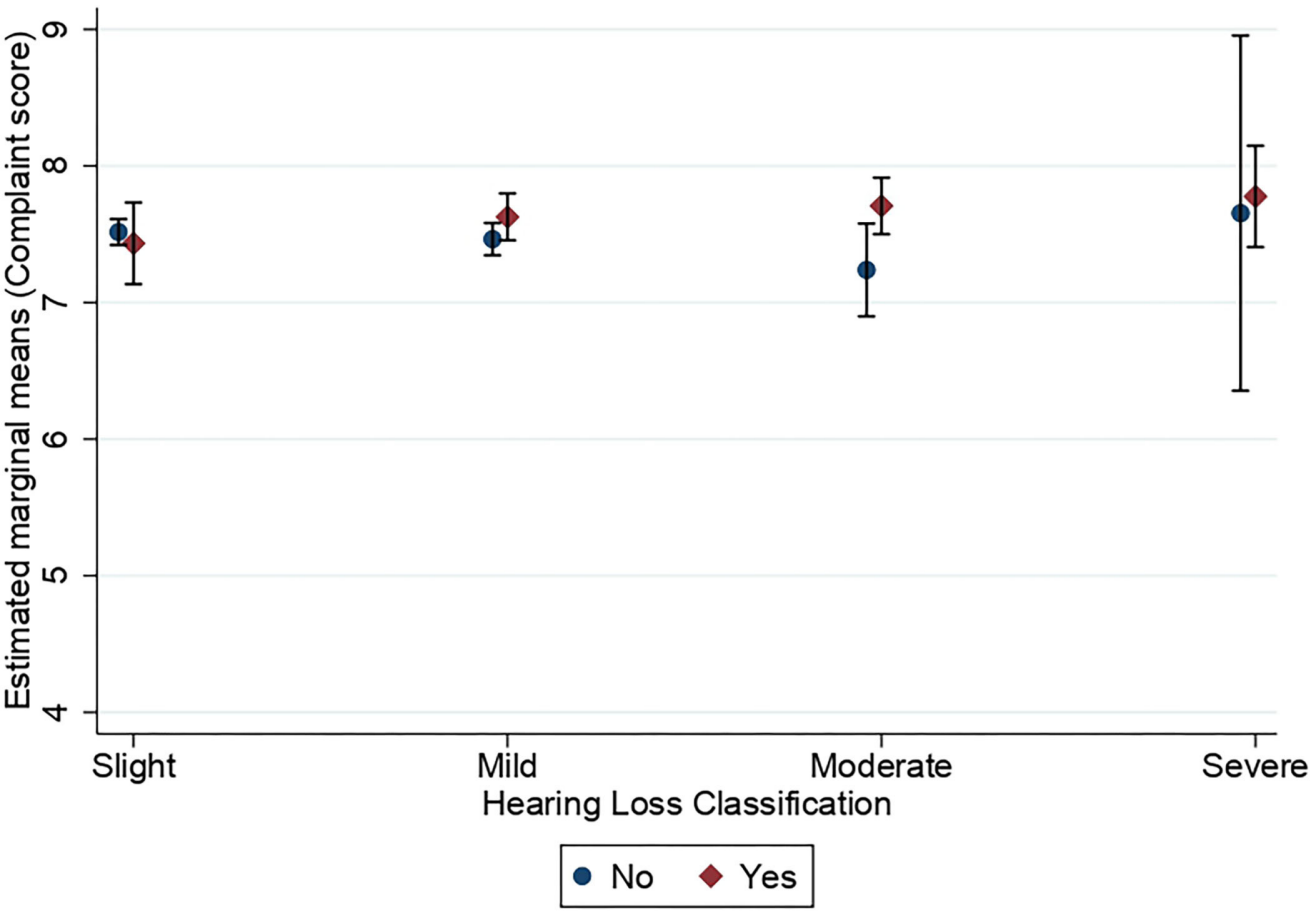
Estimated marginal means of subjective long-term memory complaints as a function of hearing status and the use of hearing aids. Estimates are averaged over the levels of age at the means of the covariates sex, education, stroke, diabetes, smoking, and hospital admission for a head injury. The error bars indicate 95% confidence intervals.

**Figure 4:**
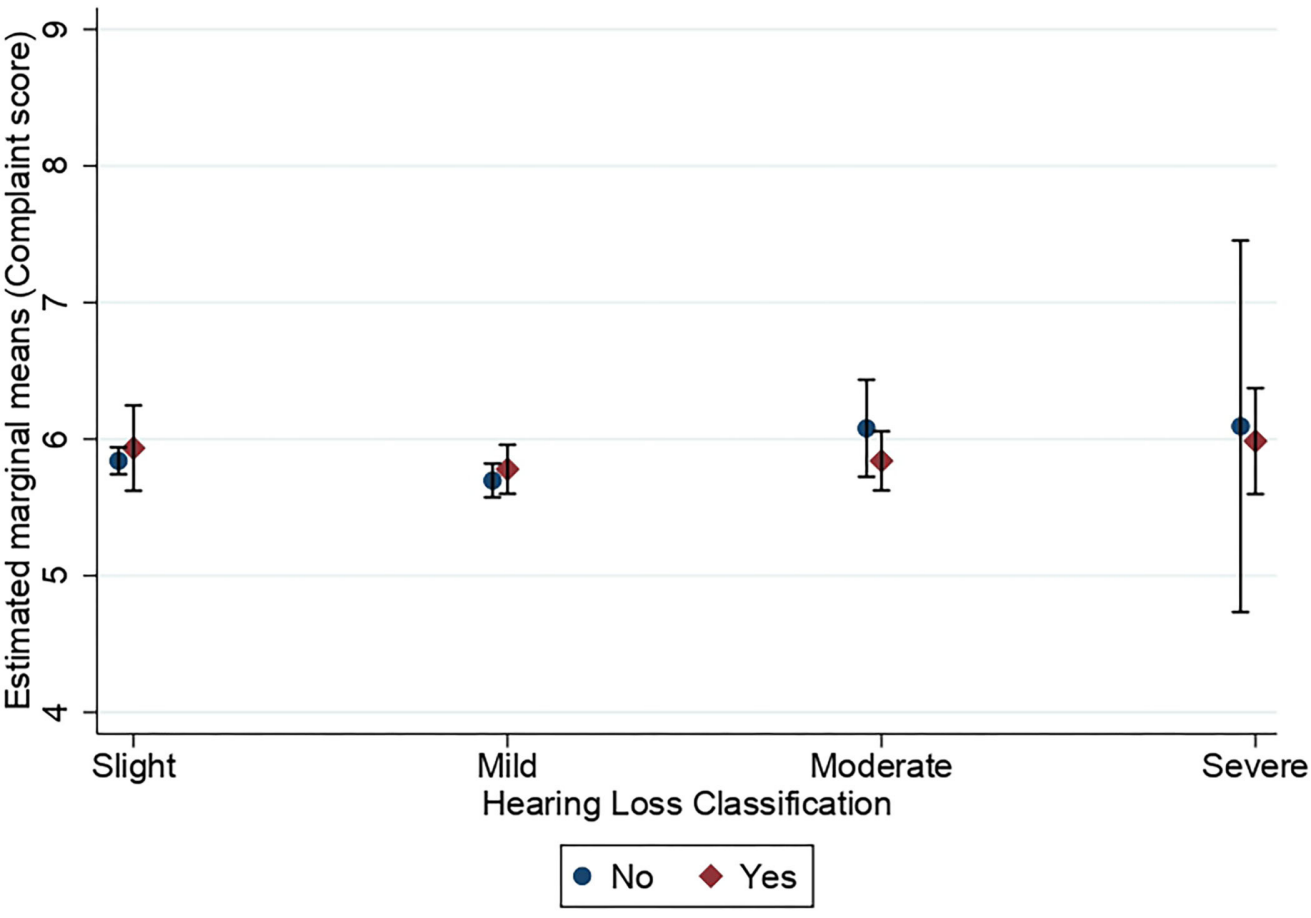
Estimated marginal means of subjective short-term memory complaints as a function of hearing status and use of hearing aids. Estimates are averaged over the levels of age at the means of the covariates sex, education, stroke, diabetes, smoking, and hospital admission for a head injury. The error bars indicate 95% confidence intervals.

Figures 3, 4 show the estimated marginal means for subjective long-term and short-term memory complaints as a function of hearing status and hearing aid use in the hearing-impaired groups. A three-way MANOVA was conducted to examine the effects of hearing status and hearing aid use on subjective long-term and short-term memory complaints in groups of people with hearing loss. The multivariate main effect for hearing status was not significant [Wilk's λ = 0.998, *F*_(6,7,714)_ = 1.46, *p* = 0.19]. However, the main effect for hearing aid use was marginally significant [Wilk's λ = 0.998, *F*_(2,3,857)_ = 3.06, *p* = 0.047, η^2^ = 0.002]. The interaction between hearing status and hearing aid use was not significant [Wilk's λ = 0.997, *F*_(6,7,714)_ = 1.63, *p* = 0.134]. A subsequent univariate ANOVA analysis showed that the main effect of hearing status was not significant for neither subjective long-term memory complaints [*F*_(3,3,858)_ = 1.42, *p* = 0.234] nor subjective short-term memory complaints [*F*_(3,3,858)_ = 1.73, *p* = 0.159]. The main effect of hearing aid use was significant for subjective long-term memory [*F*_(1,3,858)_ = 5.70, *p* = 0.017, η^2^ = 0.001] but not for subjective short-term memory [*F*_(1,3,858)_ = 3.31, *p* = 0.07]. The interaction between hearing status and hearing aid use was only significant for subjective long-term memory complaints [*F*_(3,3,858)_ = 2.76, *p* = 0.041, η^2^ = 0.002] and not for subjective short-term memory complaints [*F*_(3,3,858)_ =0.26, *p* = 0.852]. All pairwise comparisons showed no significant differences between hearing loss groups with respect to subjective long-term memory.

## 4. Discussion

Using data from a large cross-sectional study, we found that hearing loss is associated with increased subjective long-term memory complaints and not with subjective short-term memory complaints. Our findings add to the literature by showing that the negative effect of hearing loss on subjective memory complaints depends, to some extent, on the type of memory system. These findings are in line with the findings of Rönnberg et al. (10) who reported an adverse effect of hearing loss for objective long-term memory and not for objective short-term memory. The interaction between hearing aid use and hearing status on long-term memory in the hearing-impaired group is an interesting finding. One interpretation of this finding might be that as the long-term memory system is susceptible to hearing loss, this memory system can benefit from hearing rehabilitation by hearing aid. This interpretation calls for future research to further assess the effects of hearing aid use on different memory systems in people with hearing loss.

Curhan et al. (13, 14) showed a negative effect of self-reported hearing loss on subjective memory complaints. Our study, by using objective hearing measures, extends the literature by showing that the association between hearing loss and subjective memory complaints depends greatly on the type of subjective memory system. Jayakody et al. (15) reported no association between 4PTA hearing thresholds and subjective memory complaints. We reason that one explanation for this inconsistency between our findings and Jayakody et al. (15) might be that the sample size in this study was larger than Jayakody et al., which enabled us to detect small associations between different types of hearing ability and types of subjective memory complaints.

Several possible mechanisms have been hypothesized to explain the link between hearing loss and memory function. Short-term memory has a limited capacity to carry out operations for encoding, storage, rehearsal, and subsequent recall of information. Rönnberg et al. (28), in their ease of language understanding model (ELU model), proposed that working memory is employed continuously to reconstruct meaningful speech signals from less clearly heard speech signals, to map them onto corresponding phonological and lexical representations in long-term memory. In the ELU model, working memory has a dual function *to combine* speech cues that are distributed across time and frequency to finally infer meaning from the incoming speech signal. Thus, working memory is an active memory system in language understanding in people with hearing loss which subsequently results in less or no deterioration of working memory due to hearing loss.

Regarding long-term memory, the ELU model (28) assumes that the reconstruction of input signals by working memory is not always successful. Failed reconstructions of speech signals by working memory minimize the successful encoding of communicated words, meanings for lexical items, and events into episodic long-term memory. Consequently, this reduces the use of episodic long-term memory by people with hearing loss, resulting in the deterioration of episodic long-term memory in those individuals, associated with less practice and usage. In addition, the ELU model hypothesized that the mismatch between the impoverished speech signal and corresponding phonological/lexical representations, due to failed reconstructions, results in relatively less use or even disuse of semantic long-term memory in people with hearing loss. This decreased use or disuse of semantic long-term memory in people with hearing loss adversely affects the integrity of phonological and lexical representations and processing in the mental lexicon in people with hearing loss.

The strength of the present study lies in the large sample of participants in a population-based study that provides sufficient power to detect small associations between various types of hearing status and types of subjective memory complaints. In addition, the present study was the only one that used objective hearing measures to assess the hearing status of participants to evaluate the association between hearing status and subjective memory complaints. Controlling cofounders that likely biased the results was the other strength. One important limitation of the present study is that the duration of hearing aid use was not collected in HUNT4. We assume that the duration of hearing aid use plays a critical role in the association between hearing loss severity and subjective memory complaints in people with hearing loss. We encourage future studies to include the duration of hearing aid use on the association between hearing loss and subjective or objective memory functioning in people with hearing loss. The duration of hearing loss is another possible factor in the association between hearing loss and subjective memory complaints. Unfortunately, the data regarding the duration of hearing loss were not available in the HUNT 4. The present study, however, is a cross-sectional study that limits the inference of causality. Future longitudinal studies are needed to determine the direction of causality between hearing loss and subjective memory complaints and also the extent to which hearing aid use affects subjective memory complaints in people with hearing loss. Furthermore, as noted in the Method section, the participation rate was quite low at 43% which may limit the generalizability of the results. In addition, there may be potential confounders (like genetic factors) that were not included in our study. Another limitation of the current study is the small effect sizes found for the main effects of hearing loss on subjective long-term memory complaints. This may suggest that other factors associated with aging are contributing to a decrease in subjective memory complaints in people with hearing loss.

## 5. Conclusion

This study indicates an association between hearing loss and subjective long-term memory complaints and not for subjective short-term memory complaints. An interaction between hearing status and hearing aid use on subjective long-term memory was observed in hearing-impaired groups, which demands future research attention.

## Data availability statement

The data analyzed in this study was obtained from the Trøndelag Health Study (the HUNT Study), the following licenses/restrictions apply: Access to the datasets is subject to approval by the Principal Investigators of the HUNT Study. Requests to access these datasets should be directed to the HUNT Study, kontakt@hunt.ntnu.no.

## Ethics statement

The studies involving human participants were reviewed and the study was approved by the regional committee for medical and health research ethics and The Norwegian Data Protection Authority. The patients/participants provided their written informed consent to participate in this study.

## Author contributions

All authors listed have made a substantial, direct, and intellectual contribution to the work and approved it for publication.
